# Hereditary Hypofibrinogenemia with Hepatic Storage

**DOI:** 10.3390/ijms21217830

**Published:** 2020-10-22

**Authors:** Rosanna Asselta, Elvezia Maria Paraboschi, Stefano Duga

**Affiliations:** 1 Department of Biomedical Sciences, Humanitas University, Via Rita Levi Montalcini 4, Pieve Emanuele, 20090 Milan, Italy; elvezia_maria.paraboschi@hunimed.eu (E.M.P.); stefano.duga@hunimed.eu (S.D.); 2 Humanitas Clinical and Research Center, IRCCS, Via Manzoni 56, Rozzano, 20089 Milan, Italy

**Keywords:** fibrinogen, *FGG* gene, hypofibrinogenemia, storage disease, hereditary hypofibrinogenemia with hepatic storage, mutation, hepatic inclusion, prevalence

## Abstract

Fibrinogen is a 340-kDa plasma glycoprotein constituted by two sets of symmetrical trimers, each formed by the Aα, Bβ, and γ chains (respectively coded by the *FGA*, *FGB*, and *FGG* genes). Quantitative fibrinogen deficiencies (hypofibrinogenemia, afibrinogenemia) are rare congenital disorders characterized by low or unmeasurable plasma fibrinogen antigen levels. Their genetic basis is represented by mutations within the fibrinogen genes. To date, only eight mutations, all affecting a small region of the fibrinogen γ chain, have been reported to cause hereditary hypofibrinogenemia with hepatic storage (HHHS), a disorder characterized by protein aggregation in the endoplasmic reticulum, hypofibrinogenemia, and liver disease of variable severity. Here, we will briefly review the clinic characteristics of HHHS patients and the histological feature of their hepatic inclusions, and we will focus on the molecular genetic basis of this peculiar type of coagulopathy.

## 1. Fibrinogen and Related Disorders

### 1.1. Brief Overview on Biosynthesis, Structure, and Function of Fibrinogen

Fibrinogen is a 340-kDa glycoprotein circulating in plasma as a covalently linked hexamer composed of two sets of symmetrical trimers, each formed by the Aα, Bβ, and γ chains. The three chains are coded by the *FGA*, *FGB*, and *FGG* genes [1], clustered on chromosome 4q31.3. The three transcripts are mainly expressed in the liver, though appreciable levels can also be observed in stomach, lungs, kidney, and testis [2] (Supplementary Figure S1).

**Figure 1:**
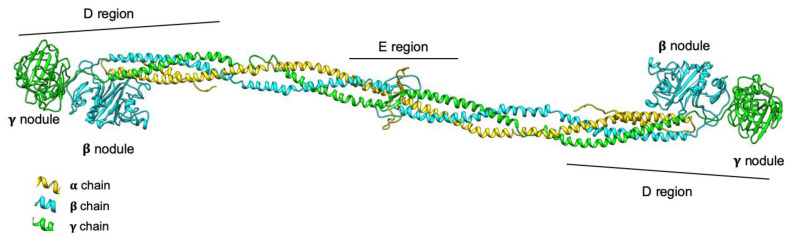
3D model representation of the fibrinogen protein. The three fibrinogen chains (Aα, Bβ, and γ) are represented in yellow, cyan, and green, respectively; the domains composing the molecule are also shown. The D regions correspond to lateral globular parts containing the C-terminus of Bβ and γ chains; the E region is the central nodule, containing the N-terminus of all chains. The model was obtained using the pdb structure 3GHG and the UCSF Chimera package.

The structure of hexameric fibrinogen is characterized by a central region (called E, containing the N-terminus of all chains) and two lateral globular regions (D, containing the C-terminus of Bβ and γ chains), connected together by coiled-coil triple helix structures (Figure 1) [3,4,5]. Fibrinogen assembly has been classically described as a step-wise process characterized, at the beginning, by the simultaneous production of Aα-γ and Bβ-γ dimers; subsequently, the third chain is added, and Aα-Bβ-γ half-molecules dimerize to form the final hexameric molecule [6]. More recently, a slightly different two-step model has been proposed, based on the incorporation of the Bβ chain into pre-formed Aα-γ complexes to constitute the trimer, followed by the fast integration of the two trimers to form the final hexamer [7]. The assembly takes place in the endoplasmic reticulum (ER), where chaperone proteins facilitate the process. In particular, the ER-resident lectin chaperones calnexin (CNX) and calreticulin (CRT) seem to be responsible for the ER retention the Aα-γ complex, acting through mono-glucosylated N-linked glycans. This allows Bβ chains to be incorporated into the Aα-γ complex immediately. The protein disulfide isomerase homologue ERp57 is instead responsible for the final assembly step of the two fibrinogen trimers into the hexamer [7]. After maturation through the Golgi network of N-linked oligosaccharides, hydroxylation, sulfation, and phosphorylation of specific residues, the mature fibrinogen is secreted into the circulation [6].

In the bloodstream, fibrinogen and its derivative fibrin (collectively fibrin[ogen]) play a central role in hemostasis, specifically in clot formation and stabilization; however, it is well known that fibrin(ogen) also participates to cell and matrix interactions, wound healing, inflammation, angiogenesis, and even in neoplastic processes [8]. In clot formation, circulating fibrinogen is converted into insoluble fibrin. Fibrin formation is determined by thrombin-mediated proteolytic removal of the N-terminal fibrinopeptides from the Aα and Bβ chains. Newly exposed α- and β-knobs insert directly into the corresponding a- and b-holes in the γ and β C-terminal regions of the D nodule of another fibrin monomer: This event allows the association of single fibrin monomers into initial protofibrils. In turn, protofibrils aggregate into fibers, thus yielding a fibrin meshwork that is fundamental for clot stability [3,9,10]. Presence of procoagulants (principally thrombin), anticoagulants, fibrin(ogen)-binding proteins, metal ions, and blood flow are factors strongly influencing the formation of the clot, as well as its structure and stability [11]. In particular, higher concentrations of thrombin are associated with the production of dense networks of highly branched fibrin fibers (which are more resistant to fibrinolysis), whereas lower thrombin concentrations determine the formation of coarse networks of unbranched fibrin fibers (which are less resistant to fibrinolysis) [12]. Importantly, fibrinogen exhibits self-assembly properties in aqueous solutions in the absence of thrombin: The lower the ionic strength (independently from the salt composition), the faster is the self-assembly, a process that takes place in a step-growth manner, where any intermediate formed cluster can coalesce with any other cluster [13].

### 1.2. Fibrinogen-Related Inherited Disorders

In healthy subjects, fibrinogen circulates in blood at concentrations ranging from 150 to 450 mg/dL; however, since fibrinogen is an acute-phase protein, during inflammation its plasmatic concentrations can exceed the level of 700 mg/dL [11,14]. Fibrinogen concentrations in plasma tend to increase with age, with women normally showing higher levels of the protein [15], a finding that is paralleled by higher levels of *FGA*, *FGB*, and *FGG* transcripts in hepatocytes [2] (Supplementary Figure S2).

Those individuals with plasma fibrinogen levels below 150 mg/dL are usually suffering from a form of acquired or inherited fibrinogen deficiency. Concerning congenital defects, these are traditionally subdivided in type I (quantitative) and type II (qualitative) deficiencies [16,17,18,19,20]. Type I deficiencies include afibrinogenemia and hypofibrinogenemia, which are respectively characterized by the lack or by reduced amounts of immunoreactive fibrinogen (<150 mg/dL), and lead to hemorrhagic manifestations that can be very mild (or even absent) or extremely severe [18]. As for type II deficiencies, the limit of 150 mg/dL is not so clear-cut: These defects comprise dysfibrinogenemia and hypo-dysfibrinogenemia, in which there are normal (or reduced) immunoreactive fibrinogen levels associated with disproportionately low functional activity values; patients who show these conditions are usually asymptomatic, more rarely they can suffer from bleeding events, thrombophilia, or both [17,18]. Type I and type II deficiencies are both determined by mutations affecting one of the fibrinogen genes: Mutations are present in the heterozygous condition in the case of hypofibrinogenemia and dys/hypodysfibrinogenemia, and in the homozygous/combined heterozygous condition for afibrinogenemia [19,20,21].

Rare cases of hypofibrinogenemia are associated with liver disease, which is caused by the accumulation of mutant fibrinogens within hepatic cells. This condition has been named hereditary hypofibrinogenemia with hepatic storage (HHHS), fibrinogen storage disease (FSD), as well as hepatic fibrinogen storage disease (HFSD); it is usually determined by heterozygous mutations leading to an impaired secretion of the mutant fibrinogen, which however maintains its ability to polymerize and aggregates spontaneously within the ER of hepatocytes.

Here, we will briefly review the molecular mechanisms leading to HHHS, with a particular focus on the mutational spectrum of the disease, which is characterized by the presence of mutations almost exclusively located in the γ chain gene.

## 2. Hereditary Hypofibrinogenemia with Hepatic Storage (HHHS)

### 2.1. Brief Historical Notes on HHHS

HHHS is a disorder belonging to the group of ER storage diseases (ERSDs), which have been recognized as an independent entity in 1987: These diseases are inborn errors of metabolism involving secretory proteins, characterized by hepatocellular storage in the rough ER and plasma deficiency of the relevant protein [22]. ERSDs comprise alpha-1-antitrypsin, fibrinogen, and alpha-1-antichymotrypsin deficiencies, and their diagnosis basically stems on immunohistochemistry and electron microscopy techniques. The storage is exclusively related to the mutant protein, whose accumulation in the ER strongly predisposes to the development of chronic liver disease of variable severity, both in children and adults [23].

The first HHHS case was described in 1981: A 30-year-old German man with a chronic hepatitis B virus infection presented with an abnormal coagulation test, which was later disclosed to be caused by low fibrinogen levels [24]. At liver biopsy, besides the typical changes due to hepatitis, the patient showed cytoplasmic proteinaceous droplets that were immunoreactive to antihuman-fibrinogen IgG. Electron microscopy investigations revealed that protein inclusions were composed of densely packed tubules of 40-nm diameter. Due to the hypofibrinogenemia observed in the patient, the morphological findings were interpreted as the direct consequence of a secretion impairment of the fibrinogen molecule. Importantly, hypofibrinogenemia was recurrent in the family, with both the father and the daughter of the proband being hypofibrinogenemic [24].

A couple of years later, a second case was reported. The patient again showed hypofibrinogenemia associated with hepatocellular inclusions similar to those previously described; hypofibrinogenemia was recurrent in the family, and an autosomal dominant transmission was postulated [25].

Later on, with the description of additional cases, it appeared clear that patients could be classified in two subsets: Those showing fibrinogen inclusions concomitantly with both the coagulation defect and the liver disease (e.g., hepatitis, primary biliary cholangitis), and those characterized by similar hepatocyte depositions but in the presence only of a liver disease. In this frame, a milestone was posed by Callea and colleagues [23] through the examination of 700 consecutive unselected liver biopsies: They identified a total of eight patients (1.15%) with fibrinogen storage (as revealed by immunohistochemistry), all affected by hepatitis (with or without cirrhosis). Among these eight patients, four also showed reduced fibrinogen levels. Overall, these figures testify from one hand the rarity of HHHS, but from the other encourage to check for plasma fibrinogen levels in the case of a cryptogenic hepatitis or cirrhosis.

Finally, the molecular basis of HHHS started to be elucidated in 2000, with the description of the first genetic defect in a family from Brescia (Italy) with hypofibrinogenemia and fibrinogen storage (see below for details) [26].

### 2.2. Histologic Characteristics of HHHS Inclusions

Fibrinogen inclusions in the ER have been traditionally classified, based on their morphological and ultrastructural features, into three different groups, called type I, II, and III [23]. Type I are inclusions of polygonal shape, with irregular outlines, corresponding to the dilated cisternae of the rough ER: These are occupied by compactly packed tubular structures, organized in fingerprint-like curved bundles. Type II are inclusions with a ground glass appearance: At electron microscopy analysis, they show granular or even fibrillar material filling ER dilated cisternae. Finally, type III inclusions share features with both the type I and II groups: They are characterized by round eosinophilic globules bordered by a clear halo, with central tubular structures similar to the type I and peripheral granular parts comparable to the type II depositions [23].

More recently, a re-evaluation of HHHS inclusions was proposed by Zen and Nishigami [27] through the study of three peculiar cases of liver disease and, above all, by an in-depth review of the literature. At biopsy, the three patients all showed fibrinogen-positive ground glass changes (type II inclusions), with one also having fibrinogen-positive intracytoplasmic globules (type III inclusions), but none of them suffered from hypofibrinogenemia. A better characterization by immunostaining of both type of fibrinogen deposits in these patients revealed the presence of additional proteins, with strong positivity for the C-reactive protein and the complement 4d fragment. In addition, the Authors observed that type II fibrinogen inclusions were identical to the “pale bodies” visible in hepatocellular carcinomas. By reviewing the literature, it became evident that: (i) Type I fibrinogen deposits are strongly associated with fibrinogen mutations, hypofibrinogenemia, and familiarity for the coagulopathy (i.e., though the relevant mutation has not been disclosed, it is possible that the condition is genetically determined); whereas (ii) type II and III inclusions are immunoreactive to many different proteins and are usually associated with liver diseases not related to fibrinogen deficiency. Given the lack of association with hypofibrinogenemia, a less specific name (e.g., pale body) was proposed as more suitable for these deposits [27].

### 2.3. Clinic Characteristics of HHHS Patients

To date, only 21 index cases characterized at the molecular level, all with a diagnosis of HHHS based on immunohistochemistry and electron microscopy findings, have been described in the English literature. Table 1 lists their main clinical features [26,28,29,30,31,32,33,34,35,36,37,38,39,40,41,42,43].

From the analysis of these 21 cases, it emerges that HHHS is equally distributed between the two sexes (52.4% males and 47.6% females), with a presentation of first symptoms (usually elevation of transaminases) at young age (mean age: 13.1 ± 20.2 years). It is associated with mild and intermittent hypertransaminasemia: Mean values for alanine aminotransferase (ALT) 191 ± 119 U/L (normal values: 5–40 U/L; 4.8 fold increase in HHHS cases with respect to the upper level); mean values for aspartate aminotransferase (AST) 147 ± 97 U/L (normal values: 7–56 U/L; 2.6 fold increase in HHHS cases with respect to the upper level).

Concerning the coagulopathy, HHHS patients usually show prolonged coagulation parameters (Table 1) but no hemorrhagic manifestations nor abnormal wound healing. Interestingly, HHHS has been associated with hypo-apo-β (APOB) proteinemia in several cases [28,36,37].

As for liver involvement, patients display a tremendous variability in the severity of liver disease, going from no signs of injury, to mild/moderate liver fibrosis, up to cirrhosis, with severe clinical pictures that can be present even in children (Table 1).

Data on medical management of HHHS are sparse; carbamazepine (CBZ) and ursodeoxycholic acid (UDCA) have been demonstrated to be beneficial in some cases [29,44]. Particularly interesting are the results obtained for the management of patients with CBZ: This drug is a well-tolerated anticonvulsive treatment, known to enhance autophagy, and its efficacy seems to be related to the normalization of ALT levels [29].

### 2.4. Genetic Basis of HHHS

**Figure 2:**
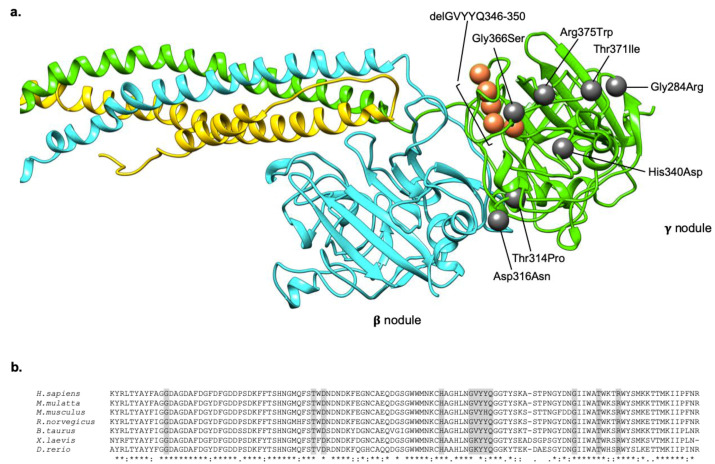
Localization and conservation of HHHS -causing mutations in the fibrinogen γ nodule. (**a**) The localization of all the mutations responsible for the HHHS phenotype, described in the literature, are indicated as grey spheres (in case of missense mutations), and orange spheres (in case of deletion). Mutations are numbered on the mature protein. The fibrinogen chains (Aα, Bβ, γ) are represented in yellow, cyan, and green, respectively. The model was obtained using the pdb structure 3GHG, and the UCSF Chimera package. (**b**) Multiple alignments of the fibrinogen γ chain terminal regions of different species. Sequences were retrieved from NCBI, and aligned using clustalW. The last line of the alignment shows in a schematic way the conservation among species. The * symbol indicates a conserved residue, the : symbol indicates conservation between groups of strongly similar properties, the . symbol indicates conservation between groups of weakly similar properties, the space indicates lack of conservation. Amino acids shaded in grey corresponds to those involved in HHHS mutations.

The genetic basis of HHHS are exclusively represented by mutations located in exons 8 and 9 of the *FGG* gene (Table 2). According to the custom, fibrinogen defects have been named after the city where the proband was coming from. Among the eight different mutations identified so far in *FGG*, seven are non-conservative missense substitutions [26,30,31,32,34,35], and one is a deletion of 14 nucleotides at the end of exon 8, leading to the activation of a cryptic splice site and the consequent production of an aberrant transcript that is predicted to be translated in a protein lacking five amino acids. [33] All mutations involve highly conserved residues localized in the C-terminal globular region of the γ chain, the so called γ module (comprising amino acids 148–411) (Figure 2). In all cases, mutations have been reported in the heterozygous state, supporting the idea that HHHS is an autosomal dominant trait and indirectly suggesting the incompatibility with life for homozygous mutations [34].

Finally, we have to mention the p.Arg35Cys missense mutation located in exon 2 of the *FGA* gene [45], which was described in a young hypofibrinogenemic patient with mild hepatomegaly and only slightly increased transaminase levels. Importantly, it has to be underlined that the identified mutation was reported in the literature as one of the most frequent cause of dysfibrinogenemia world-wide [46], directly involving the thrombin cleavage site of fibrinopeptide A, and often associated with hemorrhagic or thrombotic manifestations. Indeed, Lee and colleagues [45] claimed that they were reporting the p.Arg35Cys mutation as associated with hypofibrinogenemia for the first time (rather than with dysfibrinogenemia), but failed to report the measurement method used to evaluate fibrinogen levels in their patient. In addition, morphologic analyses were not performed with the use of specific antihuman-fibrinogen IgG, so that we are tempted to consider this specific case as a storage disorder of unknown nature, with the concomitant presence of a coagulation defect (either hypofibrinogenemia or dysfibrinogenemia).

### 2.5. Prevalence of HHHS Mutations Worldwide

The frequency of inherited fibrinogen disorders in the general population has been traditionally considered extremely low, with international registries, such as those from US, Italy, Iran, and UK, suggesting that afibrinogenemia has a prevalence of only 1–2 cases per million individuals [47]. Concerning dysfibrinogenemia and hypofibrinogenemia, these coagulopathies have always been thought to be more frequent than afibrinogenemia [48], especially considering that many patients are asymptomatic/paucisymptomatic and can go easily undetected [47]. With this in mind, we recently exploited the unprecedented power of publicly available genomic databases to define the global mutational landscape of *FGA*, *FGB*, and *FGG* as well as to determine prevalence rates of inherited fibrinogen disorders [20]. To this aim, we analyzed exome/genome data from ~140,000 individuals from the Genome Aggregation Database (GnomAD) [49], and evidenced that the world-wide prevalence for afibrinogenemia is 10-fold higher than that previously reported, and that heterozygous individuals are present in the general population at a frequency of ~1 every 100 individuals.

As for HHHS prevalence, it should be extremely rare, and only anecdotic information are available in the literature so far: (i) Aguadilla appears to be the most common mutation worldwide (with a total of 12 described index cases): (ii) *FGG* mutations associated with hepatic storage have been found in Turkey, Europe (Italy, France, Serbia), US, the Far East, the Caribbean, and Saudi Arabia (Table 1) [29,34]. With these premises, we hence checked the GnomAD for worldwide frequencies of the eight HHHS-causing mutations in the *FGG* gene (Table 2), with the hope to determine the overall prevalence rate of this particular type of hypofibrinogenemia. Unexpectedly, we did not find any of the HHHS-causing mutations reported in the literature in GnomAD, hampering us to calculate the prevalence of the disease. Considering that GnomAD contains genomic information prevalently for Caucasian individuals (58% of all analyzed DNAs belong to Caucasians vs. 42% of all other ethnicities), we also attempted to search for the eight HHHS-causing mutations in ethnic-specific databases. In particular, we explored the Greater Middle East (GME) Variome Project [50], containing data on 2497 individuals of North African or Middle East origin, and the China Metabolic Analytics Project (ChinaMAP) [51], which includes 10,588 sequenced samples of Chinese origin. Again, we were not able to find any nucleotide variation corresponding to the already reported HHHS-causing mutations. Even taking into account that we focused only on the already known genetic defects and that we could have missed so-far undisclosed mutations, our exploration of publicly available databases strongly suggests that HHHS is an ultra-rare disease (in the EU, an ultra-rare disorder is defined as affecting < 2:100,000 people [52]).

### 2.6. Molecular Mechanisms

The molecular mechanism underlying HHHS relies on the presence of mutations located in a relatively small region of the hexameric fibrinogen, i.e., in the region of the γ module between residues 310 and 401 (Table 2; Figure 2). The reasons why these mutations should cause HHHS are still not well understood, and possible explanations have been proposed, especially based on in-silico investigations.

Our research group suggested a plausible mechanism based on similarities between the structures of the γ module and of serine protease inhibitors (serpins) [31]. The γ module is a globular structure comprising three sub-domains: An N-terminal (amino acids 169–217), a central (amino acids 218–312), and a C-terminal (amino acids 313–437) domains [3,53]. The central sub-domain possesses an uncommon topology, i.e., in its five-stranded antiparallel β sheet, the central β strand is formed by the insertion of residues 407–416 that belongs to the C-terminal sub-domain. This peculiar topology resembles the one typical of serpins in their non-inhibitory forms. More in particular, serpins typically show a core domain centered on a major β sheet that constitutes the support for a mobile reactive center loop (RCL), showing the “bait” for the target protease. Upon protease cleavage, the RCL is incorporated as a sixth strand in the center of the formerly five-stranded β sheet. Consequently, the protease (which is bound in a covalent manner to the RCL), is “crushed” against the serpin, thus inhibiting the enzyme through a distortion of its active site [54]. Based on the similarities with the RCL in serpins, we speculated that also the fibrinogen γ-module β-strand insert could play functional roles through its insertion/removal from the central β sheet. Indeed, the β-strand pull-out mechanism would let the C-terminal end of the γ chain to be extended from the fibrinogen molecule, thus allowing the protein to bind to possible interactors. Importantly, it has been demonstrated that the β-strand insert can be pulled out from the central domain of the γ module without compromising its structure [55], and several observations suggest that the C-terminal part of γ module can undergo conformational changes upon conversion to fibrin or upon the binding of fibrin(ogen) with its interactors [55,56,57]. The pull-out hypothesis offers a possible explanation for the pathogenic mechanism underlying HHHS-causing mutations. In fact, it is well known that serpins are able to polymerize upon: (i) Exposure to chemicals (denaturants), (ii) mild treatment with heat, (iii) refolding from inclusion bodies, or, above all, (iv) the presence of specific missense mutations. The mechanism of such polymerization is still not completely understood, but it seems to involve the interaction of the RCL of one molecule with the β-sheet/s of another [58,59]. Similarly, the inclusion bodies typical of HHHS could be determined by perturbations related to HHHS mutations: These, by affecting the stability of the central β sheet, could allow the β-strand insert to pull out and promote the formation of fibrinogen polymers. Polymerization would be possible through the direct incorporation of the β strand into a neighboring fibrinogen molecule, or through the exposed C-terminal portion.

Overall, the above-described mechanism well reconciles with the results obtained by Burcu and collaborators [34]. They used an in-silico approach based on the calculations of protein folding free energy changes (∆∆G) between each mutant fibrinogen and the wild type counterpart. In the vast majority of cases, they observed high ∆∆Gs, indicating heavy effects on the stability of the domain (thus allowing the pull-out mechanism?). In addition, all mutations cluster near the interface of dimerization, thus reinforcing the notion that they can play a fundamental role in the polymerization process. On the other hand, these mutations, by provoking conformational changes in the region of the globular domain involved in the “end-to-end” interaction, can also cause an abnormal exposure of hydrophobic patches in the fibrinogen γ chain, which become available for interactions with lipids. This mechanism can explain the observed association of HHHS with hypo-APOB proteinemia in several patients, who were demonstrated to simultaneously accumulate fibrinogen and APOB in the same ER liver inclusions [28,36,37]. The lack of mutations in the APOB and microsomal triglyceride transfer protein (MTTP) genes in these patients further sustains the hypothesis that hypo-APOB proteinemia is as a secondary phenomenon related to *FGG* mutations and fibrinogen accumulation [28].

## 3. Open Questions and Conclusions

Though HHHS represents an ultra-rare disorder, many molecular-genetic studies were conducted and unraveled the pathomorphogenesis of the basic disease process. However, there are still questions to be answered: (i) Is it indeed true that only mutations in the *FGG* gene are predisposing to intrahepatic accumulation?; (ii) concerning phenotypic manifestations, why does the same mutation behave differently in different individuals?; (iii) why are the clinical manifestations of hypofibrinogenemia almost exclusively related to liver damage?

Many hypotheses have been put forward to explain the presence of liver disease in some mutation carriers and the absence of any liver symptom in carriers of the same mutation (even in the same family). For instance, it has been suggested that fibrinogen accumulation can be prompted by the presence of concomitant genetic defects in modifier genes, e.g., those coding for proteins taking part in protein degradation pathways. In this respect, mutant fibrinogen degradation was shown to be associated with both an ER-associated clearance and autophagy in yeast [60]. In addition, the naturally occurring surplus of fibrinogen Aα–γ assembly intermediates was demonstrated to be cleared by selective autophagy in human liver carcinoma cells (HepG2) [61]. These data, together with the observed efficacy of CBZ in normalizing ALT levels in HHHS patients [29], point to autophagy as the main pathway responsible for intracellular fibrinogen clearance, and hence suggest genes involved in the autophagic process as candidate modifiers for predisposition to fibrinogen storage.

Besides possible genetic modifiers, it has been suggested that the tendency of mutant fibrinogens to aggregate could also depend on xenobiotic intake (e.g., estrogen therapy, alcohol abuse), chronic or acute viral infections, or be secondary to cancer [62,63,64]. Among these possible triggers, acute viral infections appear particularly intriguing for two reasons: (i) Considering the young age of presentation of first liver symptoms in HHHS patients (see above), it is not probable that estrogen therapy, alcohol abuse, or even cancer, could play a role as triggering factors; (ii) the acute-phase inflammatory response to a virus entry in the organism determines a rapid plasmatic increase of numerous liver-derived proteins, including fibrinogen [65,66]. Hence, it can be assumed that, when a viral infection stimulates the acute inflammatory response, the subsequent over-production of fibrinogen can induce its accumulation in the already “crowded” ER of hepatic cells of HHHS-causing mutation carriers. In this frame, among HHHS index patients (Table 1), two showed either a form of acute viral infection (patient from Beograd) or a serious inflammatory condition (celiac disease; patient form Pisa).

Since acute viral infections could represent a powerful trigger for intrahepatic fibrinogen accumulation, a final thought must be devoted to the recent coronavirus disease 2019 (COVID-19) pandemic due to infection by the severe acute respiratory syndrome coronavirus-2 (SARS-CoV-2) [67]. Indeed, hepatic dysfunction has been seen in 14–53% of patients with COVID-19, particularly in those with severe disease [68]. More importantly, the most common pattern of coagulopathy observed in patients hospitalized for COVID-19 is characterized by increased levels of fibrinogen and D-dimer [69]. We observed the same elevation in COVID-19 patients hospitalized in our University Hospital (Humanitas Clinical and Research Center, Rozzano, Milan, Italy): The mean value for fibrinogen levels measured in a total of 725 subjects was 610 ± 213 mg/dL (normal range: 150–450 mg/dL) (unpublished data). Interestingly, it was recently reported the detailed description of a COVID-19 patient with an unusual form of liver disease, characterized by a ground-glass appearance of the hepatocytes resulting from the deposits of fibrinogen. This patient was not a carrier of fibrinogen mutations, rather, was characterized by very high plasmatic fibrinogen levels in a context of severe systemic inflammation [70]. In this frame, it could be interesting to measure fibrinogen levels also in COVID-19 asymptomatic/paucisymptomatic patients, and to understand if future cases of HHHS or, more in general, pathologic fibrinogen accumulation in the liver could possibly be traced back to the current pandemic.

## Figures and Tables

**Table 1:** Clinical characteristics of hypofibrinogenemia with hepatic storage (HHHS) probands with confirmed *FGG* mutations.

Mutant Fibrinogen ^1^	Country of Origin of the Index Case ^1^	Sex	Age (Years)	ALT/AST ^2^	Fibrinogen Levels (mg/dL) ^3^	Other Coagulation Parameters	Liver Disease	Reference
Brescia	Italy *	M	64	Transaminase elevation	Clauss = 20 Immunoreactive = 100	TCT = 47 sec	Cirrhosis	[26]
	Italy	F	49	n.r.	20	n.r.	Cirrhosis	[28]
	Caucasian	M	5	Elevated ALT	n.r.	n.r.	Mild liver disease	[29]
AI DuPont	US *	M	4	94/67	Functional = 47	PT = 15.3 sec INR = 1.5	Mild focal portal inflammatory infiltrate	[30]
Pisa	Italy *	F	3	169/105	Functional = 117 Antigenic = 136	APTT = 33.7 sec TCT = 48 sec	No sign of liver disease	[31]
Ankara	Turkey *	F	5.5	46/55	55	PT = 14 sec PTT = 24 sec INR = 1.2 TCT = 15 sec	No sign of liver disease	[32]
Angers	France *	F	35	73/51	Functional = 96 Antigenic = 123	PT = 18.4 sec	Severe chronic liver disease	[33]
Beograd	Serbia *	M	3	308/187	Functional = 66 Antigenic = 95	PT = 62.8 sec APTT = 38.2 sec TCT = 28.3 sec	No sign of liver disease	[31]
Trabzon	Turkey *	M	3.5	252/144	Clauss = 36.8	PT = 19.34 sec APTT = 35.7 sec INR = 1.69	No sign of liver disease	[34]
Aguadilla	Puerto Rico *	F	3	104/-	Clauss = 60	PT = 17.1 sec	No sign of liver disease	[35]
	Turkey	F	2	151/77	74	PT = 14.8 PTT = 26 sec INR = 1.27	Portal and septal fibrosis	[28]
	Turkey	F	5	223/411	48	PT = 13.4 sec PTT = 25 sec	Advanced liver fibrosis	[36]
	Italy	M	4	n.r.	43	n.r.	Septal fibrosis	[28]
	Japan	M	2	200/190	37.6	PT = 61.1% (n.v., 70–120%) APTT = 32 sec	Early cirrhosis	[37]
	Switzerland	M	61	131/109	70–80	PT = 16.5 sec INR = 1.41	No sign of chronic liver disease; portal hypertension	[38]
	Italy	M	6	280/110	Clauss = 57	PT = 49% (n.v., 70–120%) PTT = 42 sec	Chronic liver disease	[39]
	Caucasian	F	6	Elevated ALT	n.r.	PT = prolonged	Mild liver disease	[29]
	Siria	F	3	250/185	89	PTT = 40 sec INR = 1	Hepatomegaly	[40]
	Switzerland	F	4.5	125/111	Clauss = 70 Immunoreactive < 120	n.r.	Mild liver disease	[41]
	China	M	2	529.6/298.2	29	PT = 17.1 sec APTT = 42.8 sec	Portal fibrosis and mild hepatitis	[42]
	China	M	4	122/119	Clauss = 64	PT = 17.1 sec APTT = 43 sec INR = 1.38	Hepatomegaly	[43]

Patient’s characteristics are those reported at presentation. ^1^ Mutant fibrinogens have been named after the city of origin of the first-described index patient; the corresponding name of the country is indicated by an asterisk (*). ^2^ Normal ranges 5–40 U/L and 7–56 U/L, respectively. ^3^ Normal range 150 to 450 mg/dL. ALT, alanine aminotransferase; APTT, activated partial thromboplastin time; (normal values 25–35 s) AST, aspartate aminotransferase. F, female; INR, international normalized ratio (normal values 0.92–1.14); M, male; n.r., not reported; n.v., normal values; PT, prothrombin time (normal values 12.1–14.5 s); PTT, partial thromboplastin time (normal values 25–34 s); TCT, thrombin clotting time (normal values 24–35 s).

**Table 2:** HHHS mutations described in the literature so far.

Mutant Fibrinogen	Country ^1^	*FGG* Exon	Type of Mutation	Chr 4 Position ^2^	Nt Variation	Native Protein	Mature Protein	Status	Reference
Brescia	Italy	8	Missense	155,528,058	G > C	p.Gly310Arg	p.Gly284Arg	Heterozygous	[26]
AI DuPont	US	8	Missense	155,527,968	C > A	p.Thr340Pro	p.Thr314Pro	Heterozygous	[30]
Pisa	Italy	8	Missense	155,527,962	G > A	p.Asp342Asn	p.Asp316Asn	Heterozygous	[31]
Ankara	Turkey	8	Missense	155,527,890	C > G	p.His366Asp	p.His340Asp	Heterozygous	[32]
Angers	France	8	Deletion + Splicing	155,527,856–155,527,869	GTTTATTACCAAGG deletion	p.delGVYYQ 372–376	p.delGVYYQ 346–350	Heterozygous	[33]
Beograd	Serbia	9	Missense	155,526,174	G > A	p.Gly392Ser	p.Gly366Ser	Heterozygous	[31]
Trabzon	Turkey	9	Missense	155,526,159	C > T	p.Thr397Ile	p.Thr371Ile	Heterozygous	[34]
Aguadilla	Puerto Rico	9	Missense	155,526,147	C > T	p.Arg401Trp	p.Arg375Trp	Heterozygous	[35]

^1^ Country where the mutation was described for the first time. ^2^ Numbering according to UCSC Genome browser on Human Feb. 2009 (GRCh37/hg19) Assembly. Chr, chromosome; n.a., not available; Nt, nucleotide.

## Supplementary Materials

The following are available online at https://www.mdpi.com/1422-0067/21/21/7830/s1, Figure S1: Expression levels of *FGA*, *FGB*, and *FGG* transcripts in human tissues; Figure S2: Liver expression levels of *FGA*, *FGB*, and *FGG* genes in males and females according to age. Expression levels are shown as TPM (Transcripts Per Million), calculated from a gene model with isoforms collapsed to a single gene. Violin plots incorporate the median as well as 25th and 75th percentiles. Data are derived from the GTex database, and are stratified on sex and age of the analyzed subjects. Yo: years old; n: number of subjects.

## Abbreviations

Aα	A alpha polypeptide chain
ALT	Alanine Aminotransferase
APOB	Apolipoprotein B
APTT	Activated Partial Thromboplastin Time
AST	Aspartate Aminotransferase
Bβ	B beta polypeptide chain
CBZ	Carbamazepine
ChinaMAP	China Metabolic Analytics Project
CNX	Calnexin
COVID-19	Coronavirus Disease 2019
CRT	Calreticulin
ER	Endoplasmic Reticulum
ERp57	Endoplasmic Reticulum Resident Protein 57 (protein disulfide isomerase homologue)
ERSD	Endoplasmic Reticulum Storage Diseases
γ	Gamma polypeptide chain
*FGA*	Fibrinogen Alpha Chain Gene
*FGB*	Fibrinogen Beta Chain Gene
*FGG*	Fibrinogen Gamma Chain Gene
FSD	Fibrinogen Storage Disease
GME	Greater Middle East
GnomAD	Genome Aggregation Database
GTex	Genotype-Tissue Expression
HHHS	Hereditary Hypofibrinogenemia with Hepatic Storage
HFSD	Hepatic Fibrinogen Storage Disease
INR	International Normalized Ratio
MTTP	Microsomal Triglyceride Transfer Protein
PT	Prothrombin Time
PTT	Partial Thromboplastin Time
RCL	Reactive Centre Loop
SARS-CoV-2	Severe Acute Respiratory Syndrome Coronavirus-2
TCT	Thrombin Clotting Time
UDCA	Ursodeoxycholic Acid
∆∆G	Protein Folding Free Energy Change

## References

[1] Mosesson M.W. (2005). Fibrinogen and fibrin structure and functions. J. Thromb. Haemost..

[2] Genotype-Tissue Expression (GTex) Database, v8. https://www.gtexportal.org/home/.

[3] Spraggon G., Everse S.J., Doolittle R.F. (1997). Crystal structures of fragment D from human fibrinogen and its crosslinked counterpart from fibrin. Nature.

[4] Yee V.C., Pratt K.P., Côté H.C., Trong I.L., Chung D.W., Davie E.W., Stenkamp R.E., Teller D.C. (1997). Crystal structure of a 30 kDa C-terminal fragment from the gamma chain of human fibrinogen. Structure.

[5] Madrazo J., Brown J.H., Litvinovich S., Dominguez R., Yakovlev S., Medved L., Cohen C. (2001). Crystal structure of the central region of bovine fibrinogen (E5 fragment) at 1.4-A resolution. Proc. Natl. Acad. Sci. USA.

[6] Redman C.M., Xia H. (2001). Fibrinogen biosynthesis. Assembly, intracellular degradation, and association with lipid synthesis and secretion. Ann. N. Y. Acad. Sci..

[7] Tamura T., Arai S., Nagaya H., Mizuguchi J., Wada I. (2013). Stepwise assembly of fibrinogen is assisted by the endoplasmic reticulum lectin-chaperone system in HepG2 cells. PLoS ONE.

[8] Laurens N., Koolwijk P., de Maat M.P.M. (2006). Fibrin Structure and Wound Healing. J. Thromb. Haemost..

[9] Yang Z., Mochalkin I., Doolittle R.F. (2000). A model of fibrin formation based on crystal structures of fibrinogen and fibrin fragments complexed with synthetic peptides. Proc. Natl. Acad. Sci. USA.

[10] Lord S.T. (2011). Molecular mechanisms affecting fibrin structure and stability. Arterioscler. Thromb. Vasc. Biol..

[11] Kattula S., Byrnes J.R., Wolberg A.S. (2017). Fibrinogen and Fibrin in Hemostasis and Thrombosis. Arterioscler. Thromb. Vasc. Biol..

[12] Wolberg A.S., Monroe D.M., Roberts H.R., Hoffman M. (2003). Elevated prothrombin results in clots with an altered fiber structure: A possible mechanism of the increased thrombotic risk. Blood.

[13] Hämisch B., Büngeler A., Kielar C., Keller A., Strube O., Huber K. (2019). Self-Assembly of Fibrinogen in Aqueous, Thrombin-Free Solutions of Variable Ionic Strengths. Langmuir.

[14] Lissitchkov T., Madan B., Djambas Khayat C., Zozulya N., Ross C., Karimi M., Kavakli K., De Angulo G.R., Almomen A., Subramanian K. (2020). Fibrinogen concentrate for treatment of bleeding and surgical prophylaxis in congenital fibrinogen deficiency patients. J. Thromb. Haemost..

[15] Zierk J., Ganslandt T., Rauh M., Metzler M., Strasser E. (2019). Data mining of reference intervals for coagulation screening tests in adult patients. Clin. Chim. Acta.

[16] Asselta R., Duga S., Tenchini M.L. (2006). The molecular basis of quantitative fibrinogen disorders. J. Thromb. Haemost..

[17] De Moerloose P., Casini A., Neerman-Arbez M. (2013). Congenital fibrinogen disorders: An update. Semin. Thromb. Hemost..

[18] Casini A., de Moerloose P., Neerman-Arbez M. (2016). Clinical Features and Management of Congenital Fibrinogen Deficiencies. Semin. Thromb. Hemost..

[19] Neerman-Arbez M., de Moerloose P., Casini A. (2016). Laboratory and Genetic Investigation of Mutations Accounting for Congenital Fibrinogen Disorders. Semin. Thromb. Hemost..

[20] Paraboschi E.M., Duga S., Asselta R. (2017). Fibrinogen as a Pleiotropic Protein Causing Human Diseases: The Mutational Burden of Aα, Bβ, and γ Chains. Int. J. Mol. Sci..

[21] Casini A., Blondon M., Tintillier V., Goodyer M., Sezgin M.E., Gunes A.M., Hanss M., de Moerloose P., Neerman-Arbez M. (2018). Mutational Epidemiology of Congenital Fibrinogen Disorders. Thromb. Haemost..

[22] Callea F., De Vos R., Pinackat J., Lowe G.D.O., Douglas J.T., Forbes C.D., Henschen A. (1987). Hereditary hypofibrinogenemia with hepatic storage of fibrinogen: A new endoplasmic reticulum storage disease, in Fibrinogen 2. Biochemistry, Physiology, and Clinical Relevance.

[23] Callea F., Brisigotti M., Fabbretti G., Bonino F., Desmet V.J. (1992). Hepatic endoplasmic reticulum storage diseases. Liver.

[24] Pfeifer U., Ormanns W., Klinge O. (1981). Hepatocellular fibrinogen storage in familial hypofibrinogenemia. Virchows Arch. B Cell Pathol. Incl. Mol. Pathol..

[25] Wehinger H., Klinge O., Alexandrakis E., Schürmann J., Witt J., Seydewitz H.H. (1983). Hereditary hypofibrinogenemia with fibrinogen storage in the liver. Eur. J. Pediatr..

[26] Brennan S.O., Wyatt J., Medicina D., Callea F., George P.M. (2000). Fibrinogen brescia: Hepatic endoplasmic reticulumstorage and hypofibrinogenemia because of a gamma284 Gly→Arg mutation. Am. J. Pathol..

[27] Zen Y., Nishigami T. (2020). Rethinking fibrinogen storage disease of the liver: Ground glass and globular inclusions do not represent a congenital metabolic disorder but acquired collective retention of proteins. Hum. Pathol..

[28] Callea F., Giovannoni I., Sari S., Guldal E., Dalgic B., Akyol G., Sogo T., Al-Hussaini A., Maggiore G., Bartuli A. (2017). Fibrinogen Gamma Chain Mutations Provoke Fibrinogen and Apolipoprotein B Plasma Deficiency and Liver Storage. Int. J. Mol. Sci..

[29] Puls F., Goldschmidt I., Bantel H., Agne C., Brocker V., Dammrich M., Lehmann U., Berrang J., Pfister E.D., Kreipe H.H. (2013). Autophagy-enhancing drug carbamazepine diminishes hepatocellular death in fibrinogen storage disease. J. Hepatol..

[30] Brennan S.O., Davis R.L., Conard K., Savo A., Furuya K.N. (2010). Novel fibrinogen mutation gammaThr-Pro (fibrinogen Al du Pont) associated with hepatic fibrinogen storage disease and hypofibrinogenemia. Liver Int..

[31] Asselta R., Robusto M., Braidorri P., Pevvandi E., Nastasio S., D’Antiga O., Perisic V.N., Maggiore G., Caccia S., Duga S. (2015). Hepatic fibrinogen storage disease: Identification of two novel mutations (p.Asp316Asn, fibrinogen Pisa and p.Gly366Ser, fibrinogen Beograd) impacting on the fibrinogen-module. J. Thromb. Hemost..

[32] Callea F., Giovannoni I., Sari S., Aksu A.U., Esendaghy G., Dalgic B., Boldrini R., Akyol G., Francalanci P., Bellacchio E. (2017). Fibrinogen gamma chain mutation (c.1096C>G p.His340Asp) fibrinogen Ankara, causing hypofibrinogenemia and hepatic storage. Pathology.

[33] Dib N., Queloin F., Ternisien C., Hanss M., Michalak S., De Mazancourt P., Rousselet M.C., Cales P. (2007). Fibrinogen angers with a new deletion (gamma GVYY 346–350 causes hypofibrinogenemia with hepatic storage. J. Thromb. Hemost..

[34] Burcu G., Bellacchio E., Sag E., Cebi A.H., Saygin I., Bahadir A., Yilmaz G., Corbeddu M., Cakir M., Callea F. (2020). Structural Characteristics in the γ Chain Variants Associated with Fibrinogen Storage Disease Suggest the Underlying Pathogenic Mechanism. Int. J. Mol. Sci..

[35] Brennan S.O., Maghzal G., Schneider B.L., Gordon R., Magid M.S., George P.M. (2002). Novel fibrinogen gamma375 Arg→Trp mutation (fibrinogen Aguadilla) causes hepatic endoplasmic reticulum storage and hypofibrinogenemia. Hepatology.

[36] Sari S., Yilmaz G., Gonul I.I., Dalgic B., Akyol G., Giovannoni I., Francalanci P., Callea F. (2015). Fibrinogen storage disease and cirrhosis associated with hypobetalipoproteinemia owing to fibrinogen Aguadilla in a Turkish child. Liver Int..

[37] Sogo T., Nagasaka H., Komatsu H., Inui A., Miida T., Callea F., Francalanci P., Hirano K., Kitamura H., Yorifuji T. (2009). Fibrinogen storage disease caused by Aguadilla mutation presenting with hypobeta-lipoproteinemia and considerable liver disease. J. Pediatr. Gastroenterol. Nutr..

[38] Rubbia-Brandt L., Neerman-Arbez M., Rougemont A.L., Male P.J., Spahr L. (2006). Fibrinogen gamma375 arg→trp mutation (fibrinogen aguadilla) causes hereditary hypofibrinogenemia, hepatic endoplasmic reticulum storage disease and cirrhosis. Am. J. Surg. Pathol..

[39] Francalanci P., Santorelli F.M., Talini I., Boldrini R., Devito R., Camassei F.D., Maggiore G., Callea F. (2006). Severe liver disease in early childhood due to fibrinogen storage and de novo gamma375Arg→Trp gene mutation. J. Pediatr..

[40] Al-Hussaini A., Altalhi A., El Hag I., AlHussaini H., Francalanci P., Giovannoni I., Callea F. (2014). Hepatic fibrinogen storage disease due to the fibrinogen gamma375 Arg→Trp mutation “fibrinogen Aguadilla” is present in Arabs. Saudi J. Gastroenterol..

[41] Casini A., Sokollik C., Lukowski S.W., Lurz E., Rieubland C., de Moerloose P., Neerman-Arbez M. (2015). Hypofibrinogenemia and liver disease: A new case of Aguadilla fibrinogen and review of the literature. Haemophilia.

[42] Chang M.H., Knisely A.S., Wang N.L., Gong J.Y., Wang J.S. (2016). Fibrinogen storage disease in a Chinese boy with de novo fibrinogen Aguadilla mutation: Incomplete response to carbamazepine and ursodeoxycholic acid. BMC Gastroenterol..

[43] Gu L., Wang B., Liu L., Gan Q., Liu X., Chen L., Chen L. (2020). Hepatic fibrinogen storage disease and hypofibrinogenemia caused by fibrinogen Aguadilla mutation: A case report. J. Int. Med. Res..

[44] Maggiore G., Nastasio S., Sciveres M. (2011). Long-term outcome of liver disease-related fibrinogen aguadilla storage disease in a child. J. Pediatr. Gastroenterol. Nutr..

[45] Lee M.J., Venick R., Bhuta S., Li X., Wang H.L. (2015). Hepatic Fibrinogen Storage Disease in a Patient with Hypofibrinogenemia: Report of a Case with a Missense Mutation of the FGA Gene. Semin. Liver Dis..

[46] The Human Fibrinogen Database. https://site.geht.org/base-de-donnees-fibrinogene/.

[47] Peyvandi F. (2012). Epidemiology and treatment of congenital fibrinogen deficiency. Thromb. Res..

[48] Orphanet, the Portal for Rare Diseases and Orphan Drugs. http://www.orpha.net/consor/cgi-bin/index.php?lng=EN.

[49] The Genome Aggregation Database (GnomAD), Version 2.1.1. https://gnomad.broadinstitute.org/.

[50] The Greater Middle East (GME) Variome Project. http://igm.ucsd.edu/gme/index.php.

[51] The China Metabolic Analytics Project (ChinaMAP). www.mBiobank.com.

[52] Sobrido M.J., Bauer P., de Koning T., Klopstock T., Nadjar Y., Patterson M.C., Synofzik M., Hendriksz C.J. (2019). Recommendations for patient screening in ultra-rare inherited metabolic diseases: What have we learned from Niemann-Pick disease type C?. Orphanet J. Rare Dis..

[53] Medved L., Litvinovich S., Ugarova T., Matsuka Y., Ingham K. (1997). Domain structure and functional activity of the recombinant human fibrinogen gamma-module (gamma148-411). Biochemistry.

[54] Gettins P.G. (2002). Serpin structure, mechanism, and function. Chem. Rev..

[55] Yakovlev S., Litvinovich S., Loukinov D., Medved L. (2000). Role of the beta-strand insert in the central domain of the fibrinogen gamma-module. Biochemistry.

[56] Tsurupa G., Yakovlev S., McKee P., Medved L. (2010). Noncovalent interaction of alpha(2)-antiplasmin with fibrin(ogen): Localization of alpha(2)-antiplasmin-binding sites. Biochemistry.

[57] Lishko V.K., Kudryk B., Yakubenko V.P., Yee V.C., Ugarova T.P. (2002). Regulated unmasking of the cryptic binding site for integrin alpha M beta 2 in the gamma C-domain of fibrinogen. Biochemistry.

[58] Ekeowa U.I., Freeke J., Miranda E., Gooptu B., Bush M.F., Pérez J., Teckman J., Robinson C.V., Lomas D.A. (2010). Defining the mechanism of polymerization in the serpinopathies. Proc. Natl. Acad. Sci. USA.

[59] Yamasaki M., Sendall T.J., Pearce M.C., Whisstock J.C., Huntington J.A. (2011). Molecular basis of α1-antitrypsin deficiency revealed by the structure of a domain-swapped trimer. EMBO Rep..

[60] Kruse K.B., Brodsky J.L., McCracken A.A. (2006). Autophagy: An ER protein quality control process. Autophagy.

[61] Le Fourn V., Park S., Jang I., Gaplovska-Kysela K., Guhl B., Lee Y., Cho J.W., Zuber C., Roth J. (2013). Large protein complexes retained in the ER are dislocated by non-COPII vesicles and degraded by selective autophagy. Cell. Mol. Life Sci..

[62] Simsek Z., Ekinci O., Cindoruk M., Karakan T., Degertekin B., Akyol G., Unal S. (2005). Fibrinogen storage disease without hypofibrinogenemia associated with estrogen therapy. BMC Gastroenterol..

[63] Marucci G., Morandi L., Macchia S., Betts C.M., Tardio M.L., Dal Monte P.R., Pession A., Foschini M.P. (2003). Fibrinogen storage disease without hypofibrinogenaemia associated with acute infection. Histopathology.

[64] Radhi J.M., Lukie B.E. (1998). Pancreatic cancer and fibrinogen storage disease. J. Clin. Pathol..

[65] Gabay C., Kushner I. (1999). Acute-phase proteins and other systemic responses to inflammation. N. Engl. J. Med..

[66] Fish R.J., Neerman-Arbez M. (2012). A novel regulatory element between the human FGA and FGG genes. Thromb. Haemost..

[67] Zhou P., Yang X.L., Wang X.G., Hu B., Zhang L., Zhang W., Si H.R., Zhu Y., Li B., Huang C.L. (2020). A pneumonia outbreak associated with a new coronavirus of probable bat origin. Nature.

[68] Jothimani D., Venugopal R., Abedin M.F., Kaliamoorthy I., Rela M. (2020). COVID-19 and the liver. J. Hepatol..

[69] Covid-19 Recourses of the American Society of Hematology (ASH). https://www.hematology.org/covid-19/covid-19-and-coagulopathy.

[70] Fraga M., Moradpour D., Artru F., Romailler E., Tschopp J., Schneider A., Chtioui H., Neerman-Arbez M., Casini A., Alberio L. (2020). Hepatocellular type II fibrinogen inclusions in a patient with severe COVID-19 and hepatitis. J. Hepatol..

