# Supplementary material for: Hereditary Hypofibrinogenemia with Hepatic Storage
Source: Int J Mol Sci. 2020 Oct 22;21(21):7830. doi: 10.3390/ijms21217830 (PMC7659954; doi:10.3390/ijms21217830)
Supplement: Supplementary file 1 [file ijms-21-07830-s001.pdf]

Supplementary material

Hereditary hypofibrinogenemia with hepatic storage

Rosanna Asselta [1,2,*], Elvezia Maria Paraboschi [1,2] and Stefano Duga [1,2]

[1] Department of Biomedical Sciences, Humanitas University, Via Rita Levi Montalcini 4, 20090 Pieve Emanuele, Milan, Italy; E-Mails: rosanna.asselta@hunimed.eu (R.A.); elvezia_maria.paraboschi@hunimed.eu (E.M.P.); stefano.duga@hunimed.eu (S.D.)

[2] Humanitas Clinical and Research Center, IRCCS, Via Manzoni 56, 20089 Rozzano, Milan, Italy; E-Mails: rosanna.asselta@hunimed.eu (R.A.); elvezia_maria.paraboschi@hunimed.eu (E.M.P.); stefano.duga@hunimed.eu (S.D.)

* Correspondence: rosanna.asselta@hunimed.eu; Tel.: +39-02-8224-5215

Content:

- Supplementary Figure 1
- Supplementary Figure 2

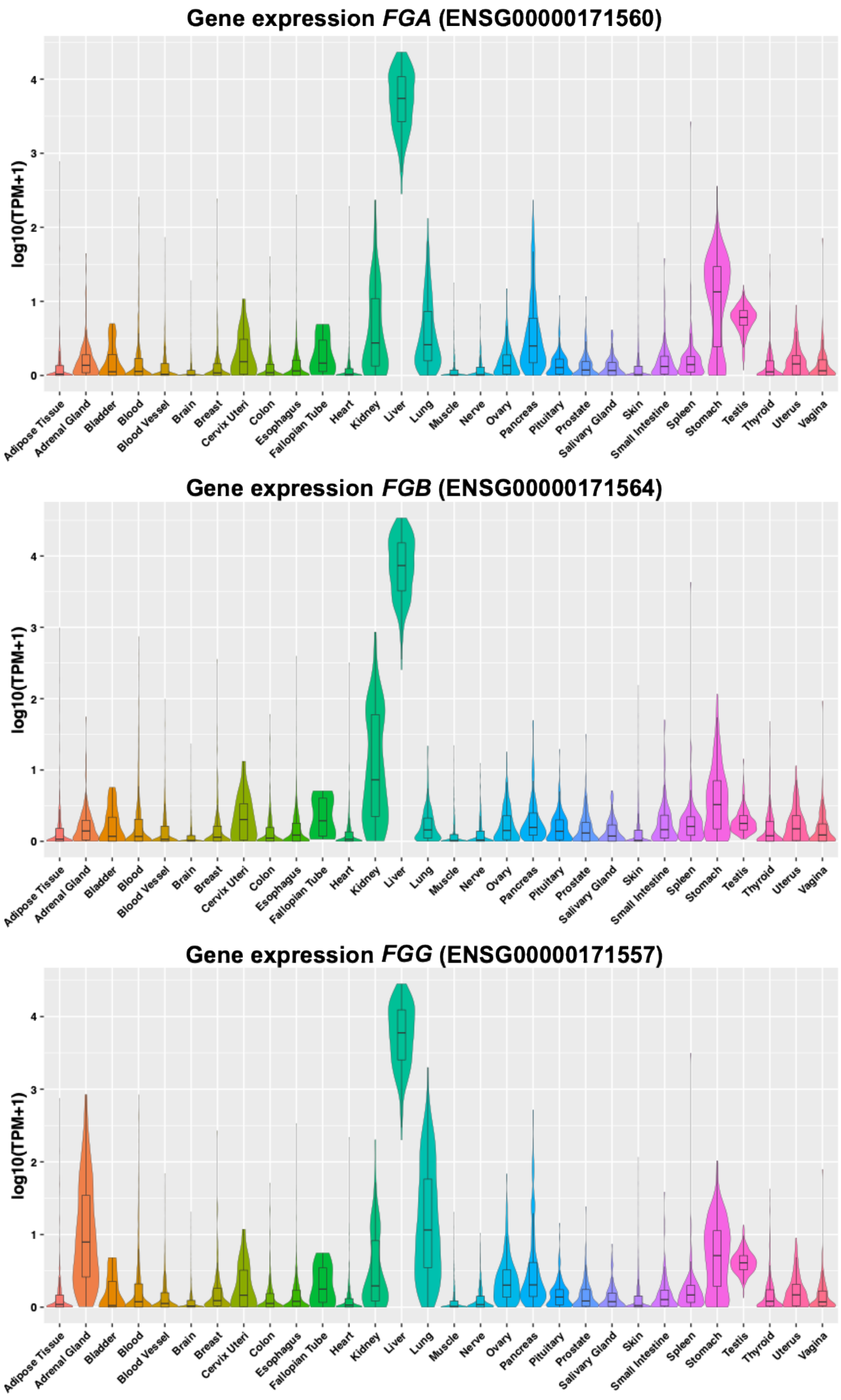

Supplementary Figure 1. Expression levels of *FGA*, *FGB*, and *FGG* transcripts in human tissues. Expression levels are shown as TPM (Transcripts Per Million; log scale), calculated from a gene model with isoforms collapsed to a single gene. Violin plots incorporate the median as well as 25th and 75th percentiles. Data are derived from the GTex database, that collected and evaluated tissue-specific gene expression from non-diseased tissue sites across nearly 1,000 individuals. Most of the sampled individuals (63.7%) had an age comprised between 50 and 69 years old, and a Caucasian origin (84.6%).

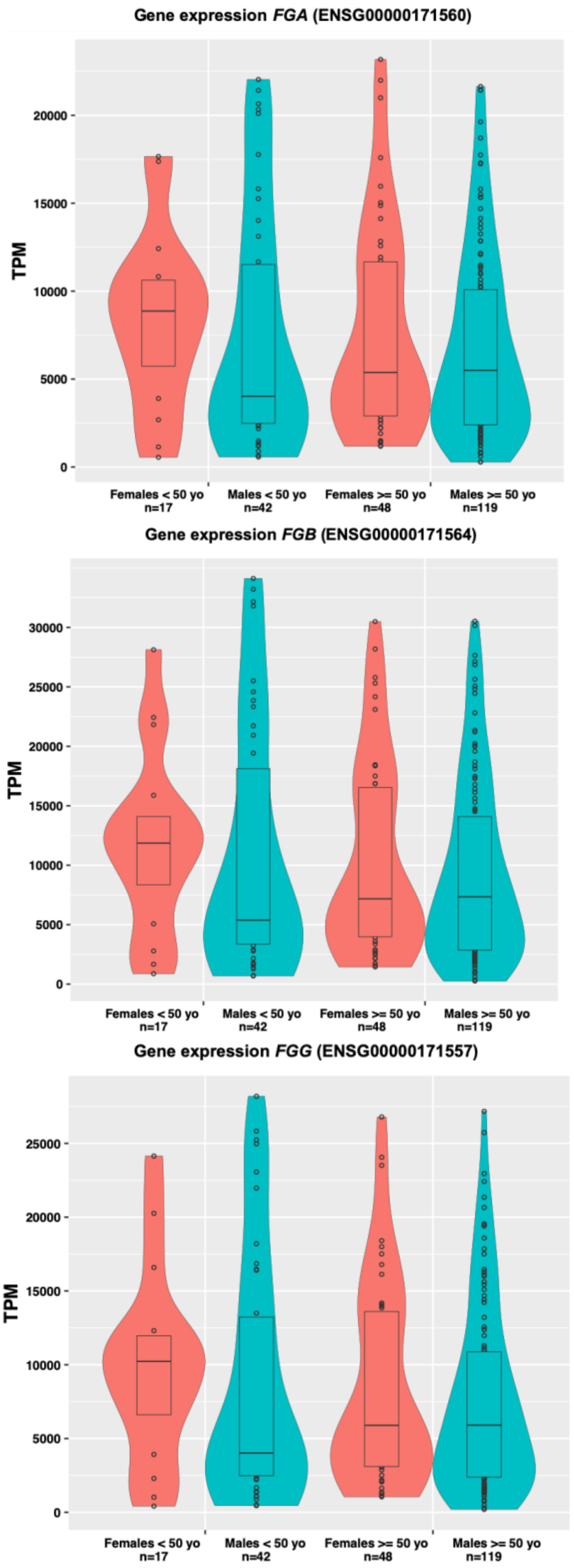

Supplementary Figure 2. Liver expression levels of *FGA*, *FGB*, and *FGG* genes in males and females according to age. Expression levels are shown as TPM (Transcripts Per Million), calculated from a gene model with isoforms collapsed to a single gene. Violin plots incorporate the median as well as 25th and 75th percentiles. Data are derived from the GTex database, and are stratified on sex and age of the analyzed subjects. Yo: years old; n: number of subjects.
